# Multiple U-Net-Based Automatic Segmentations and Radiomics Feature Stability on Ultrasound Images for Patients With Ovarian Cancer

**DOI:** 10.3389/fonc.2020.614201

**Published:** 2021-02-18

**Authors:** Juebin Jin, Haiyan Zhu, Jindi Zhang, Yao Ai, Ji Zhang, Yinyan Teng, Congying Xie, Xiance Jin

**Affiliations:** ^1^ Department of Medical Engineering, Wenzhou Medical University First Affiliated Hospital, Wenzhou, China; ^2^ Department of Gynecology, Shanghai First Maternal and Infant Hospital, Tongji University School of Medicine, Shanghai, China; ^3^ Department of Gynecology, Wenzhou Medical University First Affiliated Hospital, Wenzhou, China; ^4^ Department of Radiotherapy Center, Wenzhou Medical University First Affiliated Hospital, Wenzhou, China; ^5^ Department of Ultrasound Imaging, Wenzhou Medical University First Affiliated Hospital, Wenzhou, China; ^6^ Department of Radiation and Medical Oncology, Wenzhou Medical University Second Affiliated Hospital, Wenzhou, China

**Keywords:** automatic segmentation, U-net, ultrasound images, radiomics, ovarian cancer

## Abstract

Few studies have reported the reproducibility and stability of ultrasound (US) images based radiomics features obtained from automatic segmentation in oncology. The purpose of this study is to study the accuracy of automatic segmentation algorithms based on multiple U-net models and their effects on radiomics features from US images for patients with ovarian cancer. A total of 469 US images from 127 patients were collected and randomly divided into three groups: training sets (353 images), validation sets (23 images), and test sets (93 images) for automatic segmentation models building. Manual segmentation of target volumes was delineated as ground truth. Automatic segmentations were conducted with U-net, U-net++, U-net with Resnet as the backbone (U-net with Resnet), and CE-Net. A python 3.7.0 and package Pyradiomics 2.2.0 were used to extract radiomic features from the segmented target volumes. The accuracy of automatic segmentations was evaluated by Jaccard similarity coefficient (JSC), dice similarity coefficient (DSC), and average surface distance (ASD). The reliability of radiomics features were evaluated by Pearson correlation and intraclass correlation coefficients (ICC). CE-Net and U-net with Resnet outperformed U-net and U-net++ in accuracy performance by achieving a DSC, JSC, and ASD of 0.87, 0.79, 8.54, and 0.86, 0.78, 10.00, respectively. A total of 97 features were extracted from the delineated target volumes. The average Pearson correlation was 0.86 (95% CI, 0.83–0.89), 0.87 (95% CI, 0.84–0.90), 0.88 (95% CI, 0.86–0.91), and 0.90 (95% CI, 0.88–0.92) for U-net++, U-net, U-net with Resnet, and CE-Net, respectively. The average ICC was 0.84 (95% CI, 0.81–0.87), 0.85 (95% CI, 0.82–0.88), 0.88 (95% CI, 0.85–0.90), and 0.89 (95% CI, 0.86–0.91) for U-net++, U-net, U-net with Resnet, and CE-Net, respectively. CE-Net based segmentation achieved the best radiomics reliability. In conclusion, U-net based automatic segmentation was accurate enough to delineate the target volumes on US images for patients with ovarian cancer. Radiomics features extracted from automatic segmented targets showed good reproducibility and for reliability further radiomics investigations.

## Introduction

Ovarian cancer remains the second most common gynecological malignancy and the leading cause of death in women with gynecological cancer (1). Several imaging modalities, such as computed tomography (CT), ultrasonography (US), positron emission tomography (PET), and magnetic resonance imaging (MRI) have been used as diagnostic and treatment assessment tools for gynecological cancer all over the world (2, 3). US is a well recognized and most common applied image modality for diagnosis and assessment of ovarian cancer due to its advantage characteristics of non-invasive, no radiation, cheap and affordable (4, 5). Recently, the emerging radiomics to find association between clinical characteristics and qualitative and quantitative information extracted from US images, has further expanded the application and importance of US images for gynecological cancer (6).

By converting medical images into quantitative information, which was then analyzed subsequently using conventional biostatistics, machine learning techniques, and artificial intelligence (7), radiomics has been developed rapidly for clinical application to promote precision diagnostics and cancer treatment (8, 9). Multiple processes, such as imaging acquisition, region of interests (ROIs) segmentation, image feature extraction, and modeling, were involved in the radiomics analysis, in which ROI segmentation is the most critical, challenging, and contentious step (7).

Segmentation is the step of extracting or distinguishing a ROI from its background. It is a common and crucial stage in the quantitative and qualitative analysis of medical images, and usually it is one of the most important and earliest steps of image processing (10). Due to the low contrast, speckle noise, low signal noise ratio and artifacts inherently associated with ultrasound images, it presents unique challenges for the analysis on US images, especially for accurate segmentation of different structures and tumor volumes compared with other image modalities, e.g., CT, MRI (11, 12). The image quality of US has a high intra- and inter-observer variability across different institutes and manufactures. It also highly depends on the abundance and experience of operators or diagnosticians. All these render manual segmentation more variable and significantly impact the quantitative (e.g., radiomics) and geometric analyses with US images (13, 14).

The US segmentation problems have been the hot research topics and rapidly evolved over the past few years (11). Currently, no golden standard for tumor segmentation had been established and manual segmentation is usually applied (15). However, except for the inter and intra varieties mentioned above, the manual segmentation is also quite time consuming and boring. More recently, automatic segmentation techniques based on deep learning have become a main stream and show significant improvement in image classification predictions and recognition tasks (16). A well-known U-net architecture for biomedical imaging segmentation (17), which built uponfully convolutional network (18), has been successfully adapted to segment US images of breast (19), arterial walls (20), and gynecological cancer (21). Studies reported that the reproducibility and reliability of radiomics features could be deeply affected by the segmentation methods for CT (22), MR (23), and PET images (24). However, few studies have reported the reproducibility and stability of US based radiomics features obtained in oncology.

Previously, the feasibility of radiomics based on US images to predict the lymph node status for patients with gynecological cancer had been investigated (6). The purpose of this study is to investigate the accuracy of automatic segmentation algorithms based on multiple U-net models and their effects on radiomics features from US images for patients with ovarian cancer.

## Materials and Methods

### Patients and Images

Patients with ovarian cancer underwent radical hysterectomy and transvaginal US diagnosis at authors’ hospital from January 2002 to December 2016 were retrospectively reviewed in this study. The US images were acquired with a transvaginal ultrasonography using Voluson-E8 (GE Healthcare, Wilmington, USA) at 5–9 MHz, Philips (ATL HDI 5000, Netherland) at 4–8 MHz, and Esaote (MyLab classC) at 3–9 MHz or Hitachi (HI Vison Preirus) (Hitachi Ltd, Tokyo, Japan) at 4–8 MHz. All the images were reviewed with a Picture Archiving and Communication Systems (PACS).

Manual segmentation of target volumes was contoured by a radiologist with 7 years of experience in gynecological imaging and was further confirmed by a senior radiologist with > 15 years of experience in gynecological imaging. This retrospective study was approved by the Ethics Committee in Clinical Research (ECCR) of authors’ hospital (ECCR#2019059). ECCR waived the need of written informed consent for this retrospective study. Patient data confidentiality was confirmed.

### Automatic Segmentation Models

In this work, the classical U-net scheme and its multiple variations were used for the automatic segmentation task. Generally, the U-net is a symmetrical U-shaped model consisting of an encoder-decorder architecture (17). The left side encoder is a down-sampling used to get feature map, similar to a compression operation, while the right side decoder is an up-sampling used to restore the encoded features to the original image size and to output the results. Skip-connection was added to encoder-decoder networks in order to concatenate the features of high- and low-level together (17). When Resnet is used as a fixed feature encoder to deepen the layers of the network and solve the vanishing gradient, the U-net structure is changed to U-net with Resnet as the backbone (U-net with Resnet) (25). Resnet34 was preferred in this study.

**Figure 1:**
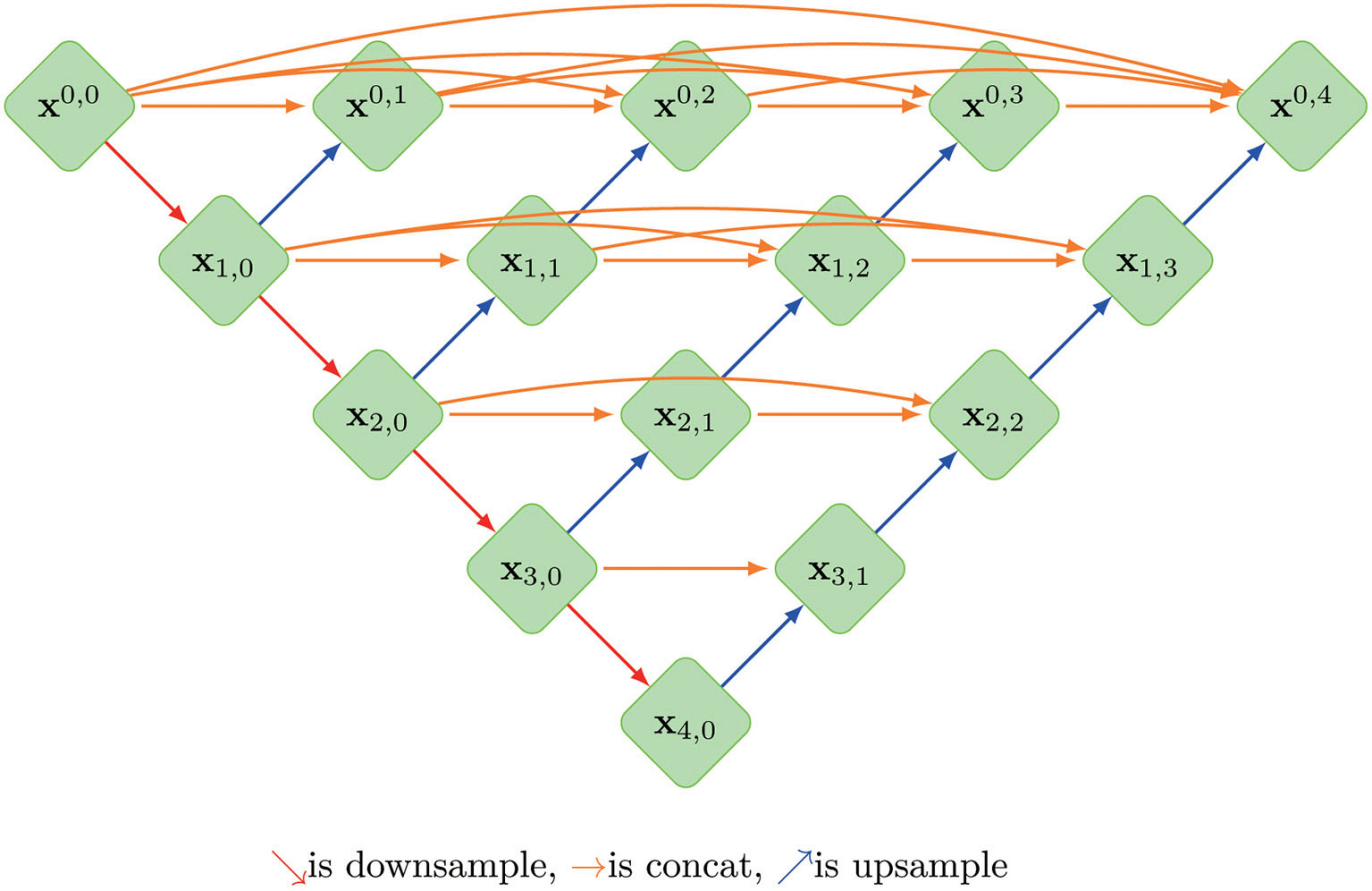
The architecture of a typical U-net model, where X^i, j^ is the operation of convolution block; Every X^i, j^(j>0)’s input is concatenated from the up-sampling of X^i+1, j-1^ from the previous convolution layer of the same dense block and all of X^i, k^(k<j) from same pyramid level.

A so-called context encoder network (CE-Net) was also employed in this study, which consists of three major parts: a feature encoder module, a feature decoder module and a context extractor. In CE-Net, Resnet block is used as a fixed feature extractor; a residual multi-kernel pooling (RMP) block and a dense atrous convolution (DAC) block consist of the context extractor module (26). U-net++ is a modified U-net with deeply-supervised encoder-decoder network, in which a series of nested, dense skip pathways are applied to connect the encoder and decoder sub-networks (27). A typical U-net structure was shown in **Figure 1**.

### Image Preprocessing

**Figure 2:**
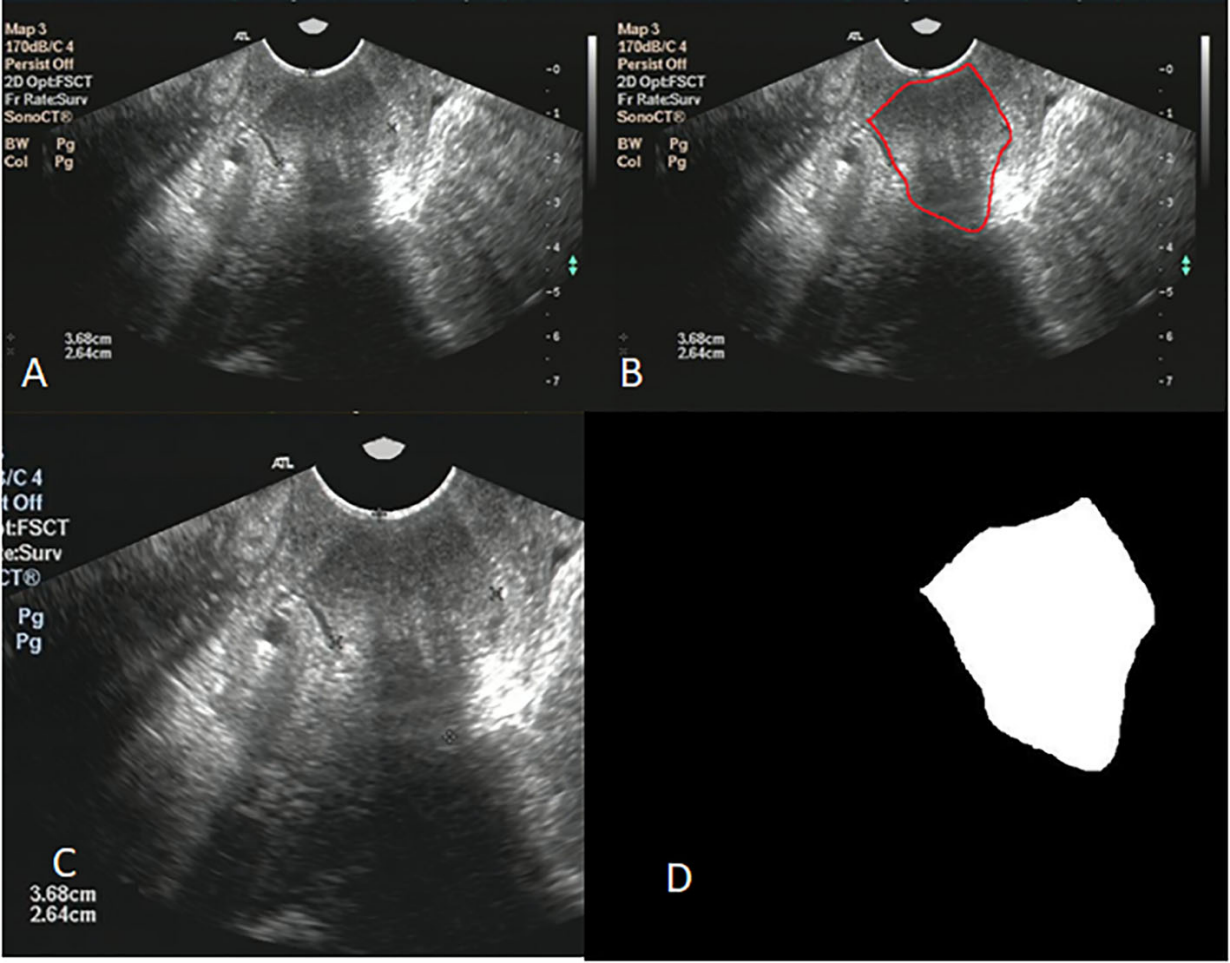
**(A)** shows the original ultrasound image; **(B)** shows ovarian tumor segmented by radiologist; **(C)** shows the image after clipping; **(D)** shows the mask of ovarian.

Image clipping was performed on each image set in order to satisfy the size requirement of U-net and to shift the center of clipping box so as to make the training model robust (28). The tumor center minus the offset (a number from 360 to 0 at -60 intervals) was selected as the starting point for a 480 * 512 clipping box. The clipping box should not exceed the image edge. A typical image preprocessing was shown in **Figure 2**.

### Radiomics Feature Extraction

After manual and automatic segmentations, the arbitrary gray intensity values on US images were transformed into a standardized intensity range by intensity normalizing. A python 3.7.0 and package Pyradiomics 2.2.0 were used to extract radiomic features from the segmented target volumes. According to different matrices capturing the spatial intensity distributions with four different scales, 79 texture features and 18 first-order histogram statistics were extracted from neighborhood gray-level different matrix (NGLDM), gray level co-occurrence matrix (GLCM), grey-level zone length matrix (GLZLM), and gray-level run length matrix (GLRLM).

### Evaluation and Statistical Analysis

The automatic segmentation models were built with the image dataset randomly divided into training sets, validation sets and test sets. The results of automatic segmentation models were evaluated by comparing them with manually segmented targets. Jaccard similarity coefficient (JSC), dice similarity coefficient (DSC), and average surface distance (ASD) were applied during the evaluation of delineation using the four U-net-related models with test data sets (29). The effects of segmentation on the radiomics features were evaluated with Pearson correlation coefficient and intraclass correlation coefficients (ICC), in which the agreement of a certain radiomic feature (e.g., shape features, texture features) between automatic and manual segmentation was evaluated by ICC (30). General statistical analyses were performed in SPSS Statistics (version 20.0.0). Statical significance was considered as a p< 0.05.

## Results

There were 127 patients with ovarian cancer and with transvaginal US images included in the study. The median age of these patients was 56 years old (from 23 to 80 years). A total of 469 US images were analyzed and randomly divided into three groups: training sets (353 images), validation sets (23 images), and test sets (93 images) for the building of automatic segmentation models. Detailed characteristics of patients and images were presented in **Table 1**. No significant difference among the training, validation, testing sets in terms of age, histological type, and tumor stages was observed.

**Table 1:** Clinical characteristics of enrolled patients and images.

Category		Patients characteristics	Images			
			Training sets	Validation sets	Testing sets p	
Total number		127	353	23	93	
Age (years)						0.344
	Mean	54.65	53.96	56.22	55.53	
	Median	56	54	59	56	
	Range	23~80	23~80	32~73	23~80	
	SD	11.85	11.38	11.01	9.79	
Histological types						0.679
	Epithelial	105	308	21	83	
	None epithelial	16	38	2	7	
	N.A.	2	12	1	5	
Tumor stages						0.691
	I	36	87	4	24	
	II	15	34	5	11	
	III	68	213	13	54	
	IV	3	19	1	4	
	N.A.	1	5	1	2	

Note. p value is calculated from the univariate association test between sub-groups, one-factor ANOVA for continues variables, Fisher’s exact test for categorized variables.

**Figure 3:**
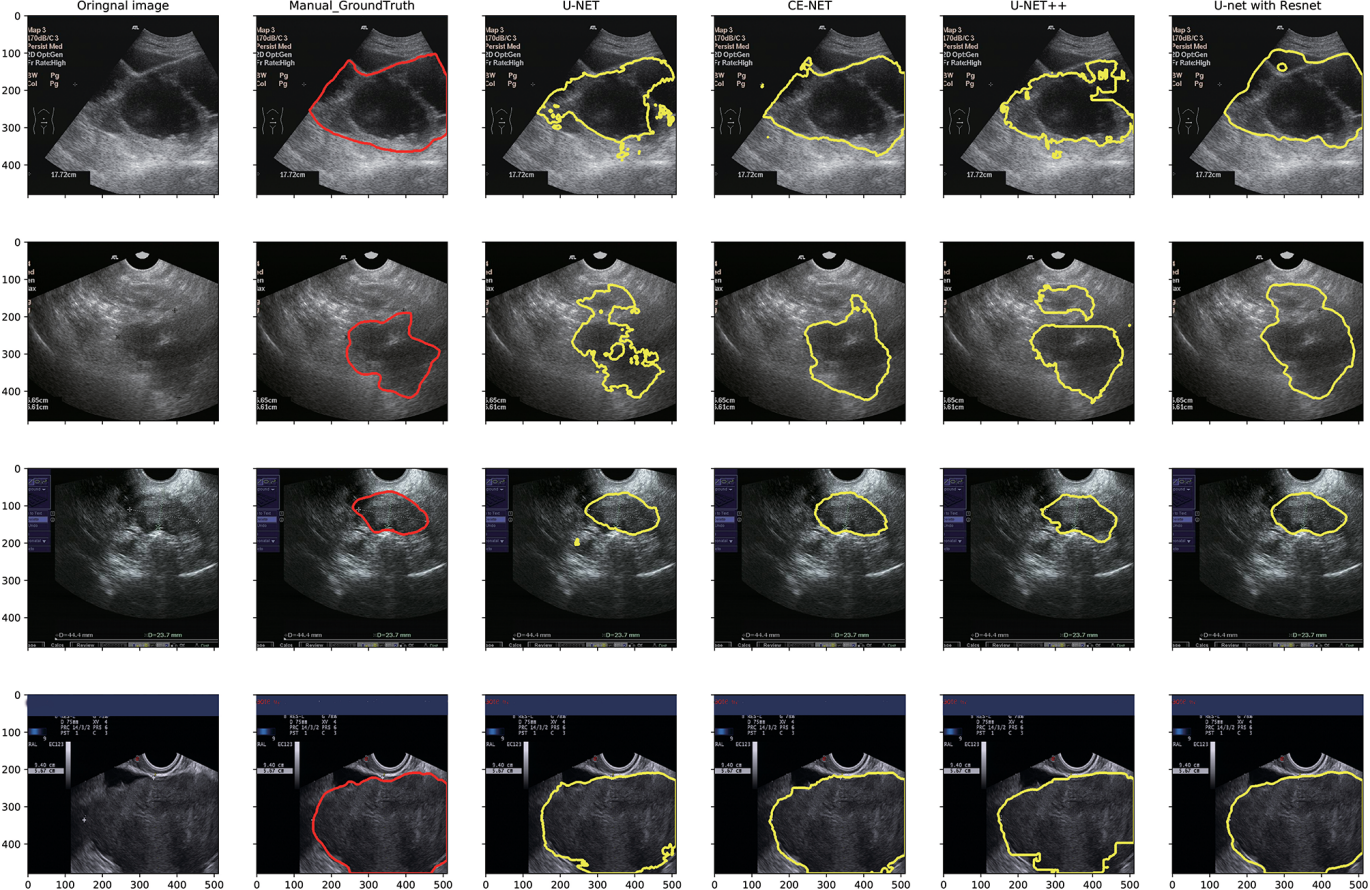
Typical segmentation results from manual delineation, U-Net, CE-NET, U-net++, and U-net with Resnet models.

U-net, CE-Net, U-net++, and U-net with Resnet were applied to delineate automatically the target volumes of ovarian cancer on US images. **Figure 3** presents typical contours achieved by these automatic segmentation models and their comparison with manual contours. Detailed results of segmentation accuracy metrics were presented in **Table 2**. CE-Net and U-net with Resnet achieved a DSC and JSC of 0.87, 0.79, and 0.86, 0.78, respectively. The ASD of CE-Net and U-net with Resnet were 8.54 and 10.00, respectively.

**Table 2:** Automatic segmentation accuracy metrics for U-net-related models.

	*Evaluation metrics*						
Models	JSC (95%CI)			DSC (95%CI)		ASD (95%CI)	
U-net	0.71		0.68~0.75	0.81	0.79~0.85	10.57	9.25~11.89
CE-Net	0.79		0.76~0.82	0.87	0.85~0.90	8.54	7.21~9.86
U-net++	0.72		0.68~0.75	0.82	0.79~0.85	10.15	8.90~11.40
U-net with Resnet	0.78		0.75~0.82	0.86	0.83~0.89	10.00	8.03~11.97

Note. JSC, Jaccard similarity coefficient; DSC, Dice similarity coefficient; ASD, Average surface distance.

**Figure 4:**
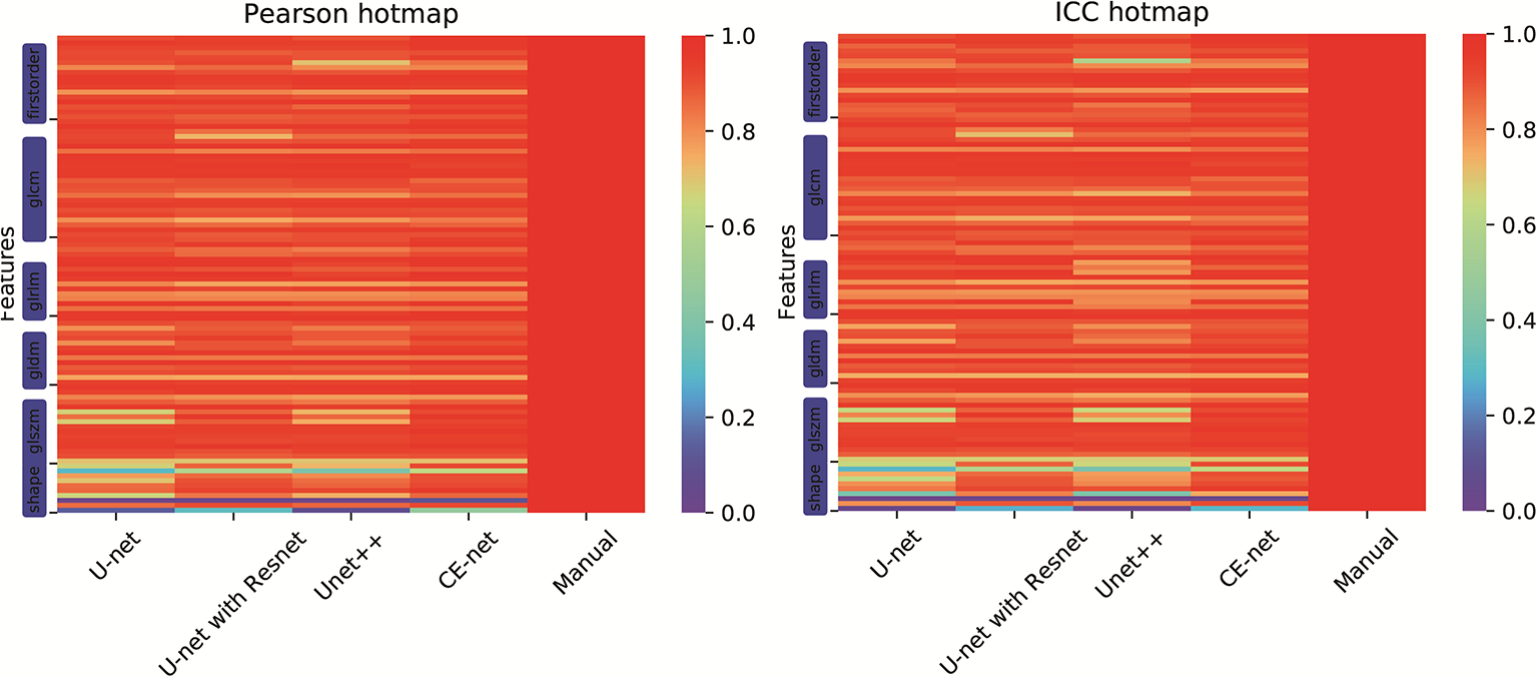
Hot maps of Pearson correlation and intraclass correlation coefficients for radiomics features extracted from manual segmentation and U-net models based automatic segmentations.

There were 97 features extracted from the delineated target volumes. **Figure 4** shows the hot maps of Pearson correlation and ICC for the comparison between features extracted from automatic segmentations and manual contours. The average Pearson correlation was 0.86 (95% CI, 0.83–0.89), 0.87 (95% CI, 0.84–0.90), 0.88 (95% CI, 0.86–0.91), and 0.90 (95% CI, 0.88–0.92) for U-net++, U-net, U-net with Resnet, and CE-Net, respectively. The average ICC was 0.84 (95% CI, 0.81–0.87), 0.85 (95% CI, 0.82–0.88), 0.88 (95% CI, 0.85–0.90), and 0.89 (95% CI, 0.86–0.91) for U-net++, U-net, U-net with Resnet, and CE-Net, respectively.

**Figure 5:**
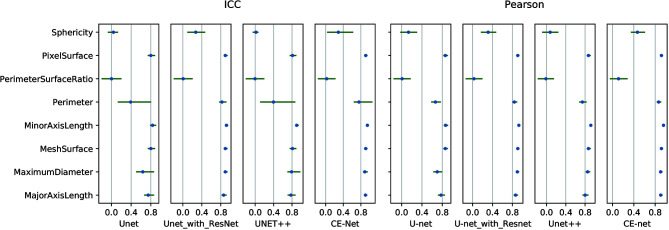
Pearson correlation and intraclass correlation coefficients for shape features extracted from different U-net automatic segmentations.

High correlations were observed for most of the features except for some features of shape GLZLM. Detailed results of Pearson correlation and ICC for all the 97 features were presented in **Supplementary Tables 1** and **2**. Further analysis on the shape GLZLM features was shown in **Figure 5**. Sphericity and PerimeterSurfaceRatio were the two shape features that showed weak correlation between automatic and manual segmentations. Excluding these two shape features, the Pearson coefficient and ICC between features extracted by CE-Net and manual segmentation ranged from 0.71–0.98, and 0.70–0.97, respectively.

## Discussion

Automatic segmentation of target volumes for ovarian cancer on US images were generated with multiple U-net models. The segmentation accuracy and its effects on radiomics features were evaluated in this study. CE-Net and U-net with Resnet models achieved a relatively higher accuracy on target delineation. Except for some shape features, most features extracted with automatic segmentation algorithms achieved high Pearson correlation and ICC in correlation with features extracted from manual contours.

US is a standard imaging modality for lots of diagnostic and monitoring purposes, had has been significantly investigated with deep learning based automatic segmentation (31). Yang et al. (32) used a fine-grained recurrent neural network to segment prostate US images automatically and achieved a high DSC around 0.92. Ghavami et al. (33) also proposed convolutional neural networks (CNNs) to automatically segment transrectal US images of prostate and got a mean DSC of 0.91 ± 0.12. Automatic segmentations on cardiac and carotid artery US images were proposed by Chen et al. (34) and Mechon-Lara et al. (35) using deep learning methods. Amiri et al. (36) fine-tuned the U-Net on breast US images and got a mean DSC of 0.80 ± 0.03. Similarly, a DSC of 0.83 to 0.90 was achieved on US images of ovarian cancer using different U-net models in this study.

U-net is a structure for medical image segmentation with superior skip connections design for different stages of the network, which had inspired the development of many variations (37). Marques et al. (21) explored different U-Net architectures with various hyperparameters in their automatic segmentations on the transvaginal US images of ovary and ovarian follicles, and indicated that architecture takes into account the spatial context of ROI is important for a better performance (21). In this study, Unet++, U-net, CE-Net, and U-net with Resnet were applied for the automatic segmentations. As shown in **Table 2**, CE-Net and U-net with Resnet exhibited higher mean DSC and JSF, and lower mean ASD compared with U-net++ and U-net, where CE-Net achieved the best performance.

In radiomics analysis, usually the ROI contoured is the region analyzed. The reproducibility and reliability of radiomics features were highly impacted by the segmentation methods. Parmar et al. (38) demonstrated that semi-automatic segmentation (ICC: 0.85 ± 0.15) provided a better feature extraction reproducibility than manual segmentation (ICC, 0.77 ± 0.17) in CT images for 20 non-small cell lung cancer patients. Heye et al. (23) achieved an ICC of 0.99 with a semiautomatic segmentation on dynamic contrast material-enhanced MR images. In this study, a highest Pearson correlation and ICC of 0.90 (95% CI, 0.88–0.92) and 0.89 (95% CI, 0.86–0.91) were achieved with CE-Net automatic segmentation. Similarly, Lin et al. (39) achieved an ICC of 0.70–0.99 on first-order apparent diffusion coefficient radiomics parameters using U-net automatic segmentation for cervical cancer.

However, a few of shape textures showed worse correlation, as shown in **Figures 4** and **5**. This may be caused by artifacts resulted from less optimal automatic segmentation algorithms as shown in **Figure 3**, which could be improved by manual correction during clinical practice. This also indicated that automatic segmentation for US images needs further investigation to improve the reliability and reproducibility of delineated volumes and radiomics features. Future evaluation of the reliability and reproducibility may be focused on prediction modeling level instead of at the level of radiomics features.

## Conclusions

U-net based automatic segmentation was accurate enough to delineate the target volumes on US images for patients with ovarian cancer. Radiomics features extracted from automatic segmented ROI showed high reliability and reproducibility for further radiomics investigations.

## Data Availability Statement

The original contributions presented in the study are included in the article/**Supplementary Material**. Further inquiries can be directed to the corresponding authors.

## Ethics Statement

The studies involving human participants were reviewed and approved by the Ethics Committee in Clinical Research (ECCR) of the First Affiliated Hospital of Wenzhou Medical University. The ethics committee waived the requirement of written informed consent for participation.

## Author Contributions

CX and XJ conceptualized and designed the study. XJ and CX provided administrative support. CX and HZ provided the study materials or patients. JJ and HZ collected and assembled the data. YA, JZ, and YT analyzed and interpreted the data. JJ and XJ wrote the manuscript. JJ, HZ, JZ, YA, JDZ, YT, CX, and XJ gave the final approval of the manuscript. All authors contributed to the article and approved the submitted version.

## Funding

This work was partially funded by the Wenzhou Municipal Science and Technology Bureau (2018ZY016, 2019) and National Natural Science Foundation of China (No.11675122, 2017).

## Conflict of Interest

The authors declare that the research was conducted in the absence of any commercial or financial relationships that could be construed as a potential conflict of interest.
